# Canadians’ knowledge of cancer risk factors and belief in cancer myths

**DOI:** 10.1186/s12889-024-17832-3

**Published:** 2024-01-30

**Authors:** E Rydz, J Telfer, EK Quinn, SS Fazel, E Holmes, G Pennycook, CE Peters

**Affiliations:** 1 School of Population and Public Health, CAREX Canada, University of British Columbia, Vancouver, Canada; 2 Department of Oncology, Cumming School of Medicine, University of Calgary, Calgary, Canada; 3 Canadian Cancer Society, Toronto, Canada; 4 Department of Psychology, Cornell University, New York, USA; 5 BC Centre for Disease Control, Vancouver, BC Canada; 6 BC Cancer, Vancouver, BC Canada

**Keywords:** Cancer, Cancer beliefs, Cancer myths, Cancer misinformation, Awareness, Thinking disposition

## Abstract

**Background:**

Many untrue statements about cancer prevention and risks are circulating. The objective of this study was to assess Canadians’ awareness of known cancer risk factors and cancer myths (untruths or statements that are not completely true), and to explore how awareness may vary by sociodemographic and cognitive factors.

**Methods:**

Cancer myths were identified by conducting scans of published, grey literature, and social media. Intuitive-analytic thinking disposition scores included were actively open- and close-minded thinking, as well as preference for intuitive and effortful thinking. A survey was administered online to participants aged 18 years and older through Prolific. Results were summarized descriptively and analyzed using chi-square tests, as well as Spearman rank and Pearson correlations.

**Results:**

Responses from 734 Canadians were received. Participants were better at identifying known cancer risk factors (70% of known risks) compared to cancer myths (49%). Bivariate analyses showed differential awareness of known cancer risk factors (*p* < 0.05) by population density and income, cancer myths by province, and for both by ethnicity, age, and all thinking disposition scores. Active open-minded thinking and preference for effortful thinking were associated with greater discernment. Tobacco-related risk factors were well-identified (> 90% correctly identified), but recognition of other known risk factors was poor (as low as 23% for low vegetable and fruit intake). Mythical cancer risk factors with high support were consuming additives (61%), feeling stressed (52%), and consuming artificial sweeteners (49%). High uncertainty of causation was observed for glyphosate (66% neither agreed or disagreed). For factors that reduce cancer risk, reasonable awareness was observed for HPV vaccination (60%), but there was a high prevalence in cancer myths, particularly that consuming antioxidants (65%) and organic foods (45%) are protective, and some uncertainty whether drinking red wine (41%), consuming vitamins (32%), and smoking cannabis (30%) reduces cancer risk.

**Conclusions:**

While Canadians were able to identify tobacco-related cancer risk factors, many myths were believed and numerous risk factors were not recognized. Cancer myths can be harmful in themselves and can detract the public’s attention from and action on established risk factors.

**Supplementary Information:**

The online version contains supplementary material available at 10.1186/s12889-024-17832-3.

## Background

Cancer is the leading cause of death in Canada [1], where two in five Canadians are expected to be diagnosed with cancer in their lifetime, and one in four is expected to die from cancer [2]. Despite decreasing cancer rates among the Canadian population, the number of cancer cases and deaths are increasing due to an aging and growing population [3, 4].

A number of risk factors can be controlled to prevent cancer cases and death, such as tobacco smoking [5], body weight [6], physical inactivity [7, 8], solar ultraviolet radiation exposure [9], inadequate vegetable and fruit consumption [10, 11], and workplace exposures [12]. In 2015, an estimated 33–37% of incident cancer cases (up to 70,000 cases) were attributable to modifiable risk factors in Canada, with tobacco smoking and low levels of physical activity contributing to the most cancer cases [13].

Similarly, death from cancer can be prevented. In 2021, an estimated 12% of Canadians over the age of 12 reported regularly smoking tobacco [14], and 29% of the Canadian population were classified as obese, with another 35% of the population classified as overweight [14]. By 2047, tobacco smoking and excess body weight are expected to be the leading risk factors for cancer death in Canada [15]. Attempts to reduce levels of overweight and obesity will need to target up-stream determinants in order to support healthy environments and behaviours [16], with care taken to reduce stigma among overweight and obese individuals.

In addition to the significant human health impacts, the economic consequences of cancer are vast. An estimated $3.3 billion of cancer management costs due to tobacco smoking from 2032 to 2044 could be avoided through prevention initiatives, as well as $3.2 billion and $2.7 billion if prevention activities were implemented to address inadequate physical activity and excess weight, respectively [17].

The 2019–2029 Canadian Strategy for Cancer Control identifies prevention as the first priority for cancer control, of which awareness is a key component [18]. However, awareness of well-established cancer risk factors has typically been low. For example, in the United Kingdom, approximately 90% and 80% of survey respondents were able to identify tobacco smoking and second-hand smoke, respectively, as risks of cancer, but fewer than a third were able to identify HPV infection and low vegetable and fruit consumption as risks [19]. Low levels of awareness of known risk factors have similarly been found in other countries, including Spain, Denmark, Sweden, and Ireland [20–22]. Overall awareness of cancer risk factors in Newfoundland and Labrador, a province in Canada, were comparatively higher [23].

In addition, cancer “myths”, which we define as including untruths or statements that are not completely true, may obfuscate and distract attention from the true risks. In the UK, approximately 40% of participants believed that stress and food additives increase cancer risk, despite weak evidence for these associations [19]. Other myths commonly believed included that non-ionizing electromagnetic frequencies (35%) and eating genetically modified foods (34%) increase cancer risk [19]. Similar beliefs were reported in the Netherlands among urinary bladder cancer survivors, although a lower percentage of people adhered to these beliefs compared to the UK [24].

Understanding who may be more susceptible to believing cancer myths and less able to identify known cancer risk factors is important when identifying appropriate audiences for cancer prevention initiatives and developing cancer prevention messages. Susceptibility to myths and other forms of misinformation emerge from an interplay of a number of factors including social, cultural, and political factors [25–29]. It can also be impacted by individuals’ thinking dispositions– how people think and make decisions [30]. For example, individuals who tend to think more analytically and in an active, open-minded way (as opposed to more intuitively) may be less susceptible to believe in myths or misinformation [31, 32].

Canadians’ knowledge of known cancer risk factors, and their beliefs in cancer myths, has not been investigated thoroughly. Assessing Canadians’ cancer knowledge and beliefs is imperative for identifying areas where awareness needs to be raised in primary prevention initiatives and health promotion activities. The purpose of this study was to apply a cross-sectional design to assess Canadians’ awareness of known cancer risk factors and selected cancer myths, and to explore potential sociodemographic and thinking disposition correlates of their cancer knowledge and myth beliefs.

## Methods

### Cancer myth identification

Multiple approaches were used to identify current cancer myths. First, the peer-reviewed literature was consulted using PubMed, Social Science Research Network, Simon Fraser University’s library database, and Google Scholar. Second, grey literature was reviewed using Google searches. Search terms used for both reviews were: (“cancer” or “causes of cancer” or “how to prevent cancer”) and (“myths” or “controversies” or “misinformation” or “misconceptions” or “fake” or “debunked”). Articles were restricted to those published in English from 2011 to 2021 that mentioned specific cancer risk factors or cancer myths. In total, 24 articles were identified through the peer-reviewed literature, and 20 articles were identified in the grey literature.

Next, two social media platforms were reviewed to identify trending cancer myths. The top 50 posts were collected for three hashtags (#causescancer, #carcinogen, and #cancercausing) from both Twitter and Instagram on July 6, 2021 (300 posts in total). Posts were included if they were related to cancer or cancer-causing agents and in English. Posts with opportunistic hash tagging or advertisement, selfies of people with cancer, and posts with no cancer content were excluded. In total, 238 (79.3%) posts were included. Peer reviewed and grey literature sources as well as social media platforms were reviewed until saturation was reached (i.e., no new topics were arising).

Finally, our formal study partners at the Canadian Cancer Society (CCS) shared data on myths that had been collected from their Cancer Information Helpline inquiries and social media, as well as resources from various international cancer organizations (12 resources in total). The resulting myths were reviewed and selected for inclusion in the survey, with additional input from partners at CCS. Those that appeared most frequently in the amassed sources, and that were of particular interest to CCS were included, with the aim of sampling a broad range of myths to best assess those that are most prevalent in Canada.

### Survey development

Similar to the CAM (Cancer Awareness Measure) and CAM-MYCS (Cancer Awareness Measure– Mythical Causes Scale) surveys, which were used in the UK to assess the public’s knowledge of known and mythical causes of cancers, respectively [19], questions were posed to assess to what extent participants agreed that the cancer risks and myths increase or decrease a person’s chance of developing cancer, with answers on a five-point Likert scale (strongly disagree, disagree, neither agree nor disagree, agree, strongly agree; note that we replaced “unsure” with “neither agree nor disagree”) [33]. In total, 31 questions on cancer myths and 19 questions on known cancer risk factors were selected for inclusion in the survey.

The survey collected demographic data, including province/territory of residence, age, gender, population density of current place of residence, racial or ethnic background, and total annual household income. The survey also captured participants’ thinking dispositions using a comprehensive thinking styles questionnaire which assesses the degree of respondents’ actively open-minded thinking (AOT), preference for intuitive thinking (PIT), preference for effortful thinking (PET), and close-minded thinking (CMT) [34]. AOT assesses the willingness to consider new information, despite personal beliefs [35]. PIT evaluates the reliance on intuition [34]. PET explores likelihood or engagement in thinking or analyzing. CMT examines black and white thinking or the scale on which truth is relative. Assessing thinking dispositions allows us to assess key correlates of beliefs to gain a better understanding of the reasoning behind why people believe what they do.

Finally, an instructional manipulation was included to assess the participants’ attention. In the survey, participants were asked to respond to the following: “I am very busy these days and do not have time to follow the latest research. To show that you’re still paying attention, answer both “strongly disagree” and “disagree”.” Participants who failed to correctly follow the prompt were excluded from the analysis. The survey is included in Additional File 1.

Surveys were administered in English using Qualtrics in November 2021. Adults aged 18 + and residents of Canada who were registered on Prolific were eligible to participate in the study. An equal number of men and women were recruited via Prolific’s participant selection criteria during survey set-up. Eligibility was confirmed using Prolific participant registration characteristics and a presurvey. Prolific participants are provided with a nominal fee based on how many minutes they spend on a survey. At the time of data collection, Prolific recommended an hourly rate of £7.50 and we estimated that the survey would take 10 min for an estimated payment of £1.25 ($2.12 Canadian dollars at the time of the survey). A random selection of cancer myths and risk factors were presented to each participant to reduce participant burden and chances of inattentiveness, especially given the similarity of the questions asked [36]. In addition, this helped to ensure that a broad sample of risk factors and myths and questions on thinking disposition could be included in the survey without undue burden on the study participants. A pilot of the study was run prior to distribution to participants.

### Analysis

All statistical analysis was run using SAS version 9.4. The breakdown of responses to each myth and risk factor was calculated and visualized. This was completed for each question overall, by specific demographic variables, and the percentage of myths and facts correctly identified were calculated. Chi-square analyses were conducted to assess the significance of differences in the percentage responding correctly. If a participant responded “agree” or “strongly agree” to a question about a known risk factor, or “disagree” or “strongly disagree” to a question about a cancer myth, they were considered correct. Correlations among thinking style subscales, and correlations between thinking style subscale scores and cancer myths and risk factor questions, were assessed using Pearson or Spearman correlation, depending on variable type.

## Results

In total, 765 surveys were completed. Those with improper Prolific IDs were excluded (*n* = 6), as were those who responded incorrectly (*n* = 18), or not at all (*n* = 2), to the attention check. Following this, only one participant had not completed the survey, who was then excluded. For participants who had completed the survey multiple times (identified through duplicate IP addresses and Prolific IDs), only the first response was retained for analysis. In total, a sample of 734 participants was included in the analyses (96%).

Participants were primarily from Ontario (48%), large cities (population from 250,000 to < 1 million) (37%), and of white ethnicity (56%) (Table 1). Participants were relatively well distributed across income categories, but the largest income group was those making $120,000 or more (18%). On average, participants were 31 years of age, with age ranging from 18 to 78 years.

Overall, respondents were better able to identify known risk factors (facts) (70% were correctly identified on average) compared to myths (49%) (Table 1). When examined by demographic variables, variations in the average percentage of participants responding correctly across known risk factors were observed by population density, with greater identification among higher density areas, and income where the highest earning individuals were better able to identify cancer risk factors relative to the other groups, but particularly relative to those earning $120,000 + and $20,000–$39,999 (74% for the high income category compared to 66% in the lower income category). Significant differences in awareness of known risk factors and also cancer myths were observed by ethnicity (p-value < 0.001), where Black participants consistently had lower risk factor and cancer myth awareness (62% and 40%, respectively) compared to other ethnicities. Differences in awareness of cancer myths were also observed by province, with Albertans having the highest awareness of myths (54%), compared to Ontario and Quebec (approximately 47%). Furthermore, age was associated with an apparent *increase* in percentage correctly identifying facts, but a *decrease* in the percentage correctly identifying cancer myths.

**Table 1:** Study sample demographics and the percentage of correctly identified myths or facts

Variable	N (%)	% correctly identified	
		Myths	Facts
**Overall**	**734**	**49.4**	**70.4**
**Province (p-value)**		**< 0.001**	**0.47**
Ontario	351 (47.8)	47.7	70.2
British Columbia	131 (17.9)	50.2	72.4
Alberta	118 (16.1)	54.1	70.2
Manitoba and Saskatchewan	58 (7.9)	49.3	69.1
Quebec	41 (5.6)	46.6	69.7
Atlantic Canada (NB, NL, NS, PE)	35 (4.8)	51.9	68.4
**Gender (p-value)**		**< 0.001**	**0.29**
Man	360 (49.1)	49.4	70.0
Woman	352 (48.0)	48.8	71.0
Other	22 (3.0)	61.8	66.4
**Population density (missing ** ***n*** ** = 2)** **(p-value)**		**0.28**	**0.026**
Metropolitan center (population of 1 million or more)	224 (30.1)	49.8	72.2
Large city (population from 250,000 to < 1 million)	272 (37.2)	48.5	70.5
Small town (population from 50,000 to < 250,000)	162 (22.1)	50.0	69.2
Rural (population of less than 50,000)	67 (9.2)	51.8	67.5
Prefer not to say	7 (1.0)	46.2	58.6
**Ethnicity (p-value)**		**< 0.001**	**< 0.001**
White	411 (56.0)	50.4	72.3
Chinese	80 (10.9)	46.3	72.0
South East Asian	50 (6.8)	52.0	66.8
South Asian	48 (6.5)	47.3	67.2
Mixed	44 (6.0)	53.3	65.1
Black	33 (4.5)	39.8	62.3
Other (includes prefer not to say)	68 (9.3)	49.6	68.5
**Income (missing ** ***n*** ** = 6)** **(p-value)**		**0.18**	**0.001**
Less than $20,000	52 (7.1)	46.8	74.1
$20,000 to $39,999	86 (11.8)	49.9	66.4
$40,000 to $59,999	112 (15.4)	52.0	71.9
$60,000 to $79,999	104 (14.3)	48.6	70.6
$80,000 to $99,999	100 (13.7)	50.7	69.7
$100,000 to $119,999	80 (11.0)	48.7	65.6
$120,000 or more	131 (18.0)	49.6	74.4
Prefer not to say	63 (8.7)	47.4	69.1
**Continuous variables**	**Mean (Range)**	**% correctly identified**	
		**Myths**	**Facts**
**Age**	31.3 (18–78)	**< 0.001**	**0.002**
18–25 (*n* = 289)		50.4	67.4
26–35 (*n* = 232)		49.5	72.1
36–45 (*n* = 129)		51.0	72.8
> 45 (*n* = 84)		43.5	72.0
**Thinking disposition scores** ^**a**^	^b^		
Close-Minded Thinking 6–10 (*n* = 154, 21.0%) 11–15 (*n* = 337, 45.9%) 16–20 (*n* = 174, 23.7%) 21–30 (*n* = 69, 9.4%)	**14.0 (6–28)** 8.4 (IQR 3.0) 13.0 (IQR 2.0) 17.7 (IQR 2.0) 22.6 (IQR 2.0)	**< 0.001** 54.3 51.1 44.0 44.8	**< 0.001** 74.5 71.2 66.4 66.9
Actively Open-minded Thinking 11–15 (*n* = 55, 7.5%) 16–20 (*n* = 198, 27.0%) 21–25 (*n* = 315, 42.9%) 26–30 (*n* = 166, 22.6%)	**22.2 (11–30)** 13.7 (IQR 2.0) 18.5 (IQR 2.0) 23.3 (IQR 2.0) 28.4 (IQR 3.0)	**< 0.001** 42.7 45.4 50.1 56.7	**< 0.001** 65.6 64.9 72.3 75.3
Preference for Effortful Thinking 10–15 (*n* = 41, 5.6%) 16–20 (*n* = 120, 16.4%) 21–25 (*n* = 390, 53.1%) 26–30 (*n* = 183, 24.9%)	**23.0 (10–30)** 13.4 (IQR 3.0) 18.6 (IQR 2.0) 23.6 (IQR 3.0) 28.5 (IQR 2.0)	**< 0.001** 46.1 45.2 50.2 51.8	**< 0.001** 66.0 65.1 70.3 77.1
Preference for Intuitive Thinking 6–12 (*n* = 85, 11.6%) 13–17 (*n* = 218, 29.7%) 18–22 (*n* = 299, 40.7%) 23–30 (*n* = 132, 18.0%)	**18.1 (6–30)** 10.3 (IQR 3.0) 15.2 (IQR 2.0) 20.3 (IQR 3.0) 24.6 (IQR 1.0)	**< 0.001** 58.1 54.3 45.9 42.6	**< 0.001** 74.6 72.7 68.9 66.3

^a^Thinking disposition scores are categorized by score

^b^Mean and range represents the mean and range of the Thinking disposition score

Results by thinking styles showed that people who identified facts and myths correctly had slightly higher mean active open-minded thinking (AOT) and preference for effortful thinking (PET) scores than those who were incorrect (Table 2). Conversely, people who identified facts and myths correctly had slightly lower mean preference for intuitive (PIT) and close-minded thinking (CMT) scores than those who were incorrect.

**Table 2:** Fact and myth identification by thinking style

Fact or myth	Correct or	Mean AOT	Mean PET	Mean PIT score
Fact	Correct	22.5 (4.29)	23.3 (3.94)	17.9 (4.44)
Fact	Incorrect	21.7 (4.35)	22.6 (4.00)	18.6 (4.32)
Myth	Correct	22.6 (4.33)	23.2 (3.95)	17.7 (4.40)
Myth	Incorrect	21.8 (4.31)	22.8 (4.01)	18.6 (4.33)

**Fig. 1:**
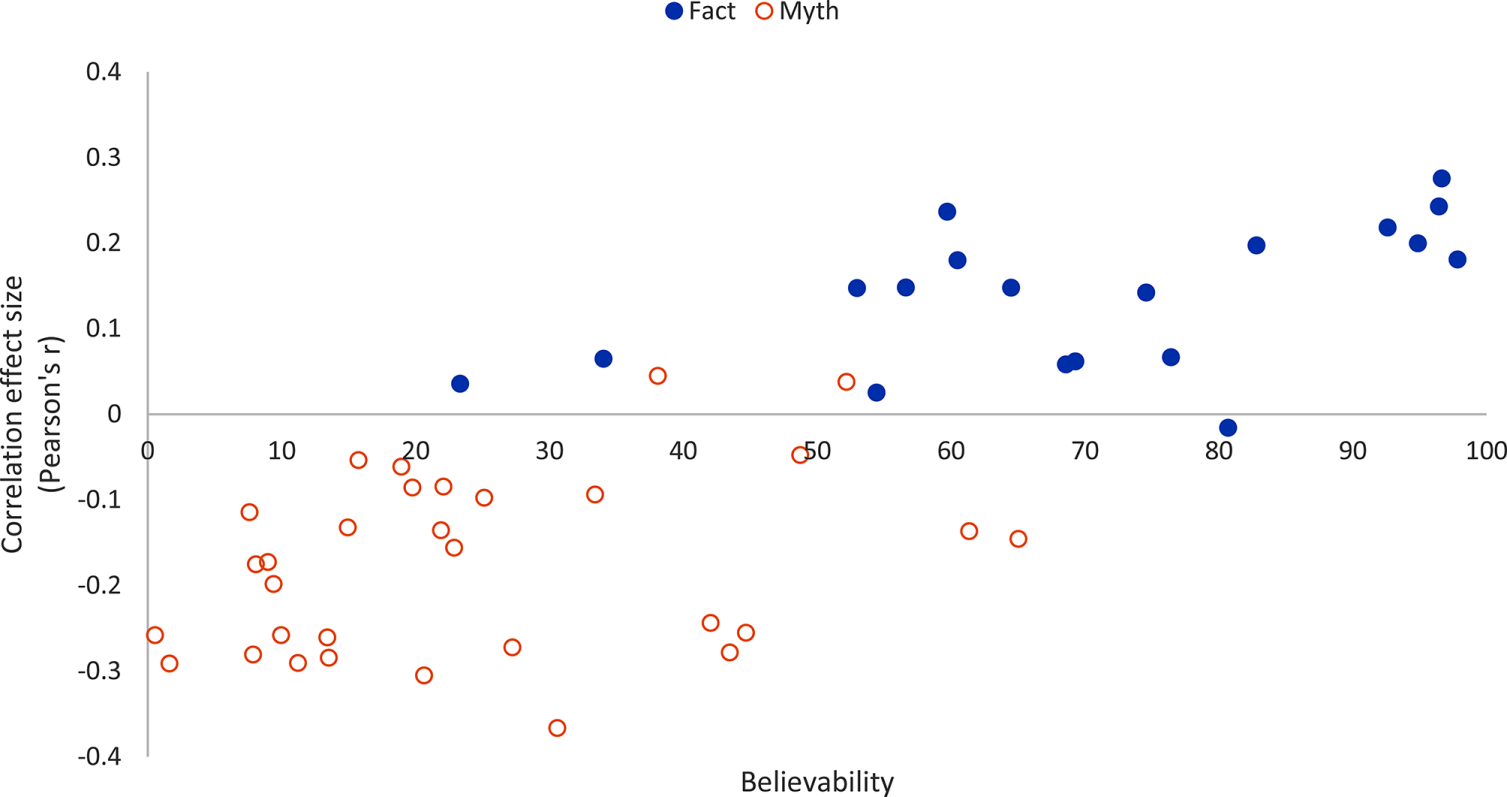
Scatterplot displaying, for each item, the correlation between overall believability (i.e. the percent agreeing or strongly agreeing with an item, y-axis) and the correlation effect size (Pearson’s r) of the global mean of the thinking disposition score global and response to specific myths and facts

We also assessed the extent to which analytic thinking correlates with believability (i.e. % agreeing or strongly agreeing with an item) for each statement and overall (Fig. 1). We found a strong and positive correlation between the believability of facts and correlation between response to the item (i.e. the fact or myth) and the global mean of the thinking disposition score (*r* = 0.78, *p* < 0.001). At the item-level, correlations tend to be more strongly negative for more unbelievable claims, and more strongly positive for more believable claims.

**Fig. 2:**
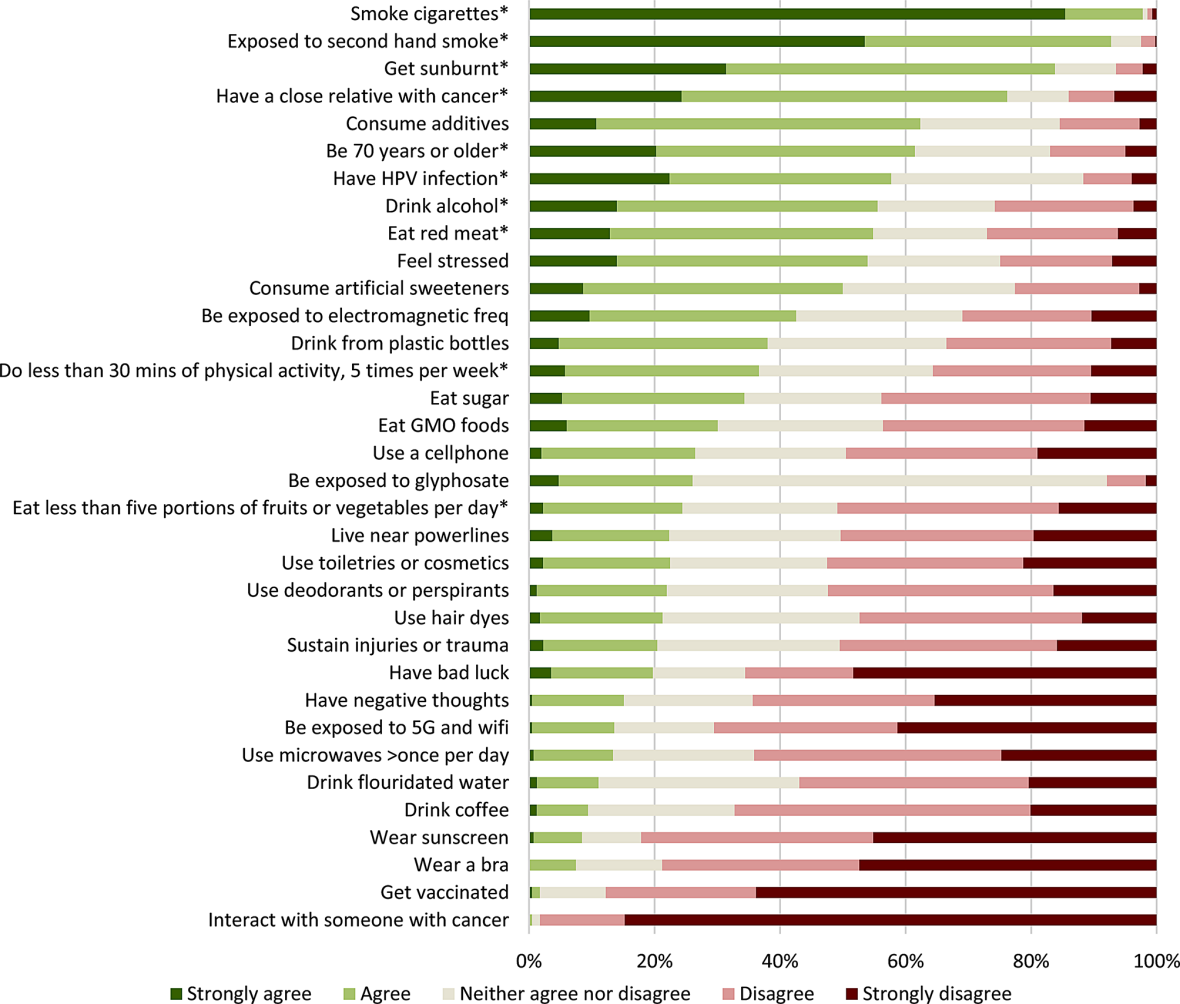
Percent breakdown of responses to “to what extent do you agree that the following can increase cancer risk”? (*n* ~ 400 per factor) *Known risk factors

Over 90% of respondents agreed or strongly agreed that tobacco-related risk factors (smoking cigarettes and being exposed to second-hand smoke) increase cancer risk (Fig. 2). However, less people were able to correctly identify the other known risk factors that increase cancer risk, including getting sunburnt (83%), having a close relative with cancer (75%), being 70 years or older (60%), having an HPV infection (57%), drinking alcohol (54%) and eating red meat (53%).

Relatively few participants correctly identified exercising less than 30 min 5 times per week (34%) and eating less than five portions of vegetables or fruit per day (23%) as cancer risk factors. Furthermore, there was considerable uncertainty for these two risk factors; approximately a quarter of participants neither agreed nor disagreed that doing less than 30 min of physical activity per day or eating less than 5 portions of vegetables or fruits increased cancer risk. Uncertainty was only higher for one other known risk factor; almost a third of participants neither agreed nor disagreed that an HPV infection increases cancer risk.

The top myths that were incorrectly identified (i.e. participants agreed or strongly agreed that they increase cancer risk, contrary to expert consensus) were consuming additives (61%), feeling stressed (52%), consuming artificial sweeteners (49%), being exposed to electromagnetic frequencies (42%), drinking from plastic bottles (38%), eating sugar (33%), and eating GMO foods (31%). Between 20 and 30% believed that using a cellphone, toiletries or cosmetics, deodorants or antiperspirants, and hair dyes, being exposed to glyphosate, living near powerlines, and having bad luck increase cancer risk. Between 10 and 20% believed that having negative thoughts, sustaining physical injuries and trauma, using microwaves more than once a day, being exposed to 5G and WiFi, and drinking fluoridated water increase cancer risk. Only a few participants (5–10%) believed that drinking coffee, wearing sunscreen, and wearing a bra increase cancer risk.

The greatest uncertainty in the cancer myths was for glyphosate exposure, where 66% of participants neither agreed nor disagreed that glyphosate increases cancer risk, followed by drinking fluoridated water (32%), using hair dyes (31%), sustaining injuries or traumas (30%), and consuming additives (28%).

**Fig. 3:**
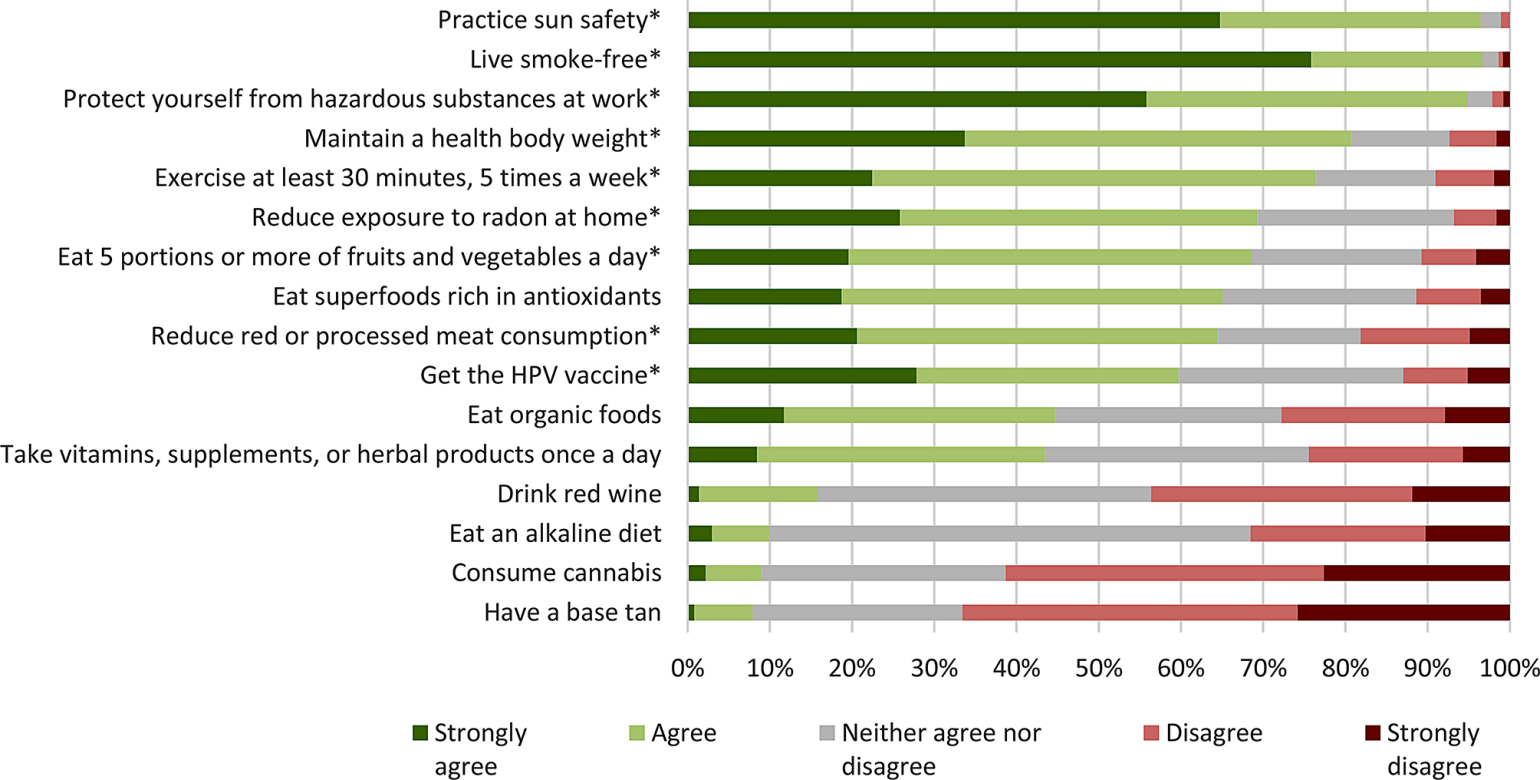
Percent breakdown of responses to “to what extent do you agree that the following can reduce cancer risk”? (*n* ~ 400 per factor) *Known protective factors

When asked about factors that reduce cancer risk (Fig. 3), participants correctly identified the following known protective factors: living a smoke-free life (97% agreed or strongly agreed), practising sun safety (96%), reducing workplace exposures (95%), maintaining a healthy body weight (81%), and exercising at least 30 min, 5 times a week (76%). The least identified known protective factors were having an HPV vaccine (with only 60% of respondents agreeing or strongly agreeing), reducing red meat consumption (64%), and reducing radon in the home (69%). Eating superfoods rich in antioxidants (with 65% believing it protects against cancer), eating organic foods (45%), and consuming vitamins and supplements (43%) were the most commonly believed myths. Only 10% agreed or strongly agreed that eating an alkaline diet protects against cancer, but almost 60% neither agreed nor disagreed with the statement, showing a high level of uncertainty. A significant proportion of respondents were also unsure whether the following myths were protective against cancer: drinking red wine (41%), consuming cannabis (30%), consuming vitamins and supplements (32%), eating organic foods (28%) and having a base tan (26%). The greatest uncertainty for known protective factors were: having the HPV vaccine (27%), reducing radon (24%), and eating 5 portions or more of vegetables or fruit in a day (21%).

## Discussion

We assessed Canadians’ awareness of known cancer risk factors and beliefs in cancer myths. Similar to previous studies that examined both myths and known risk factors [19, 37], respondents were better able to identify known cancer risk factors over cancer myths. For example, in the UK, 52% of known risk factors and 36% of cancer myths were accurately identified, compared to 70% of risk factors and 49% of myths in this study [19]. Variations in the average percentage of correctly identified cancer facts and myths were observed by demographic group, particularly for income, ethnicity, and age, consistent with previous studies [19, 23, 38, 39]. Differences in beliefs about cancer myths and risk factors by thinking style were observed, suggesting that those who are more actively open-minded and prefer effortful thinking may be better able to identify cancer myths and risk factors, while those who are more close-minded and prefer intuitive thinking are less able. When examining the correlations between the thinking styles and individual cancer myths in particular, more items were significantly correlated with actively open-minded thinking– i.e., the tendency to question beliefs according to evidence - compared to the three other thinking styles (although only small to modest correlations were observed throughout) [40, 41]. Additionally, people who were correct in their responses regarding cancer myths and facts had slightly higher mean active open-minded thinking (AOT) and preference for effortful thinking (PET) scores than those who were incorrect. People who provided correct answers had slightly lower mean preference for intuitive (PIT) and close-minded thinking (CMT) scores than those who were incorrect. The small differences in thinking styles means and relatively small effect sizes for the relationships between the thinking styles and the individual myths and facts may suggest that the scales overlap in the space of health misinformation somewhat. However, our work showed that people with higher AOT scores in particular were more consistently correct in their identification of cancer myths. This is demonstrated by the higher negative value Spearman correlation coefficients seen in Additional File 2 for AOT scores and, for example, eating GMO foods. The higher the AOT score, the less likely a respondent was to agree with the statement that GMO foods increase the risk of cancer, which is a correct interpretation. People with higher AOT scores were also more likely to correctly identify correct facts about cancer prevention, such as practicing sun safety or getting the HPV vaccine. Higher PET scores followed similar trends as the AOT scores, though the results were not as strong. Conversely, people with higher CMT and PIT scores were less likely to answer questions about facts or myths correctly. For example, one of the strongest positive correlations we detected with PIT scores demonstrated that people with higher PIT scores were more likely to believe that drinking fluoridated water, living near power lines, and eating GMO foods increase the risk of cancer. Thinking dispositions and cancer myths and risk factor knowledge, specifically, have not been studied to our knowledge; however, the broader literature has shown evidence that being actively open-minded, thinking more analytically, and having a need for cognition (or, preference for effortful thinking) are significant factors when it comes to the discernment of fake news and health misinformation [29, 30, 32, 31–44]. In other words, those that are willing to question their beliefs based on evidence, and those who enjoy and are willing to dwell and think through information are more likely to identify cancer myths. These findings may have implications on how and with whom information should be shared in the future in order to increase cancer risk factor knowledge [45]. The 4 intuitive-analytic thinking styles that we used in our study are a very new tool for research of this nature, and further use of them in the design of potential interventions is needed as their use evolves and increases [46].

Study participants had variable awareness of known cancer risk factors and cancer myths. Tobacco continues to be a well recognized risk factor for cancer (with over 90% of participants correctly identifying tobacco smoking and exposure to second-hand smoke), but less than 35% were able to correctly identify low vegetable and fruit intake (less than 5 portions of vegetables or fruit per day) and low physical activity (less than 30 min of physical activity, 5 times a week) as risk factors for cancer. These findings were consistent with results found in the UK [19]. However, in Newfoundland and Labrador, being over 70 years of age, drinking more than one unit of alcohol per day, and having a diet low in fibre were the least identified risk factors (≤ 65% correct) [23]. The results on relatively poor recognition of alcohol use and low fibre diets may not be as surprising as the fact that older age was poorly recognized as a factor; for most cancers, age is the most significant predictor of cancer risk [3]. The most commonly endorsed myths among our participants were consuming additives (61%), feeling stressed (52%), consuming artificial sweeteners (49%), and being exposed to electromagnetic frequencies (42%). These are consistent with the findings from the UK study, where the top three myths were stress (42%), food additives (41%), and exposure to electromagnetic frequencies (35%) (19). Although a different population was surveyed, these findings suggest potentially persistent beliefs in these myths across countries.

Recommendations to counter misinformation include targeting the “undecided majority” over the “unswayable minority” who hold more extreme views that are particularly resistant to change [47]. For this reason, it is important to examine the myths and risk factors with a high percentage of “neither agree nor disagree” responses. By doing so, incorrect beliefs where there is more room for further communication and for which awareness initiatives may be more successful can be identified and targeted. The greatest uncertainty for cancer myths was presented for glyphosate as a risk factor for cancer, and alkaline diets as a form of prevention (approximately 60% for both). The high degree of uncertainty may relate to real uncertainties about glyphosate exposure and cancer risk. To date, messaging and media reports on hazardous potential and/or human health risks of glyphosate remains mixed with conflicting conclusions by major agencies, including the International Agency for Research on Cancer, the European Union, and the US EPA [48–54]. We were unable to identify published literature that examines myths related to alkaline diets and cancer and that could help to explain such a high level of uncertainty in this myth, but it may suggest that people have heard of these diets but are not sure about any conclusions made on the topic. It is also possible that respondents were unsure about what these substances or practices are, and so they were less sure in their responses.

Participants were also uncertain about drinking fluoridated water, using hair dyes, and sustaining injuries or traumas as cancer risks. They were also uncertain about drinking red wine, consuming cannabis, taking vitamins and supplements, eating organic foods, and having a base tan as being protective. While some of these are mostly harmless, they may distract from known protective factors, such as increasing overall vegetable and fruit intake, or physical activity. For example, organic foods may also be prohibitively expensive for people with lower household incomes, and fear of consuming pesticides on non-organic fruits and vegetables could lead to the unintended consequence of avoidance of these foods, which would be detrimental to one’s overall health and cancer risk. Furthermore, some of these beliefs are outright harmful. Drinking any amount and type of alcohol, including red wine, increases the risk for at least 9 different types of cancer [55]. Having a base tan is not protective against skin cancer [56], and indoor tanning, which is used to acquire base tans, actually increases skin cancer risk [57]. Fluoridated water is an important public health measure that reduces the risk of dental caries among children [58], and attempts to minimize exposure to fluoridated water could negatively impact oral health while increasing disparities across socioeconomic lines as many lower income households may not be able to maintain regular dental care.

For known risk factors, approximately 20–30% of participants were uncertain whether the HPV vaccine, reducing radon exposure at home, and eating 5 portions or more of vegetables and fruits a day reduces the risk of cancer. Similarly, approximately a quarter to a third of participants were uncertain whether eating less than 5 portions of vegetables or fruit and HPV infection increases cancer risk, suggesting that social marketing campaigns to educate the public about HPV, including the HPV vaccine and how it can prevent certain types of cancer, as well as the importance of vegetable and fruit intake, could be beneficial. In particular, with nearly 100% of cervical cancers being due to HPV infection, there are concerted efforts to increase HPV immunization with the goal of eliminating cervical cancer in Canada in Canada by 2040 and these findings can be used to inform this work [59, 60]. Furthermore, radon is a known lung carcinogen and the leading risk factor for lung cancer in people who don’t smoke tobacco [61, 62], yet it appears that a large proportion of respondents do not understand the risk of radon exposure. This is especially important in Canada which has among the highest home-based exposures to radon in the world [63]. Promotional campaigns are needed now to inform the public about the risks related to radon exposure and to encourage residents to test their homes. Given that people can only make decisions within the environments that they live, campaigns should be complemented with healthy public policies and supportive environments to reduce barriers to cancer prevention [64].

Several questions were presented for their presence or absence in both fact and myth categories, such as eating or not eating five servings of fruits and vegetables a day or getting/not getting 30 min of exercise five days week. We saw that for fruit and vegetable consumption, respondents agreed that meeting the recommendations was preventive against cancer, but did not agree as much that not meeting the recommendations actually increases cancer risk. Similarly, respondents indicated high awareness that meeting exercise recommendations reduces the risk of cancer, but low awareness that not meeting the recommendations is a risk factor for cancer. These findings may offer important information on framing for cancer prevention campaigns.

This is the first national study that assessed Canadians’ knowledge/awareness of risk factors and cancer myths, with consideration of respondents’ thinking dispositions. By recruiting participants through Prolific [65], we were able to obtain a large and diverse sample of participants. Although a sex-balanced sample was achieved, the sample for this study was relatively young relative to the Canadian population. Furthermore, Prolific is prone to selection bias in terms of how Prolific is advertised, who chooses to participate in Prolific surveys [66] and which surveys are chosen for completion by participants. In order to reduce participant burden, we presented only a random selection of myths and risk factors to each participant. While this allowed us to increase our sample size, we were unable to conduct any predictive modelling for the participants’ overall awareness of cancer myths and risk factors.

Future analyses should examine individual risk factors and myths in more detail, with particular attention to the undecided majority and predictors of beliefs. In addition, Canadians’ awareness of other aspects of the cancer control continuum, including cancer treatment options should be explored, since this is an area ripe with misinformation [67, 68]. Furthermore, people with cancer have been shown to have high rates of complementary alternative medicines use [69, 70].

There were several limitations to our approach. Though our search strategy for survey design (regarding which questions to ask the participants) was broad, it did not necessarily locate all possible cancer myths and facts that are in circulation in the published and grey literature or on social media. Though the survey was developed with careful thought and reference, it was not directly evaluated for clarity/comprehension using a cognitive interview approach. We did run a small pilot of the survey before launching, but no concerns were identified about some of our questions which may have been less clear. Without a vetting process for the survey, we may have overlooked potential challenges. For example, the responses regarding glyphosate may be attributed to lack of understanding of what it is, rather than a misunderstanding of whether it causes cancer. The options did not include an “I don’t know” or “I don’t know what that is” option, so people who may have fallen in those categories had to mark “neither agree, nor disagree”. Our question about whether cancer can be attributed to “having bad luck” was included due to media coverage of a particular study several years ago that pervasively made its way into many media sources that were not well-reported, but respondents may have interpreted ‘bad luck’ to mean genetic mutations, which is indeed a true risk factor for cancer. When collecting demographic information, we only collected information on income and not highest education in order to limit the number of questions for respondents and because these two variables are often highly correlated. Therefore, it is certainly possible that income is acting as a proxy for higher education in our study and may explain why higher income respondents were more accurate at identifying cancer myths and facts. Further, for findings to be representative, it would have been beneficial to have used multiple sources for recruiting research participants to have a more diverse sample to reduce sampling bias.

In summary, we found that while Canadians were able to identify some known cancer risk factors well, many myths are believed and many risk factors are still not recognized. Larger, multi-year campaigns, such as those for smoking cessation, seem to have worked as many respondents understand the risks related to tobacco smoking. Future work should aim to increase awareness of lesser known but established risk factors, such as radon, vegetable and fruit consumption and HPV vaccination while debunking cancer myths so that evidence-based information can be prioritized. The results of this study can be used by relevant stakeholders to prioritize myths for debunking and lesser-known risk factors for social marketing campaigns with complementary shifts in effective healthy public policies.

## Electronic supplementary material

Below is the link to the electronic supplementary material.

Supplementary Material 1: Additional file 1

Supplementary Material 2: Additional file 2

Supplementary Material 3: Additional file 3

Supplementary Material 4: Additional file 4

## Data Availability

The datasets generated and/or analysed during the current study will be made available upon request with any potentially identifiable information removed (e.g., IP addresses). Additional tabulated or analyzed data are available from the corresponding author on reasonable request.

## Abbreviations

AOT: Actively open-minded thinking
CAM: Cancer Awareness Measure
CAM-MYCS: Cancer Awareness Measure– Mythical Causes Scale
CCS: Canadian Cancer Society
CMT: Close-minded thinking
GMO: Genetically modified organisms
HPV: Human papilloma virus
PET: Preference for effortful thinking
PIT: Preference for intuitive thinking
