# Correction: Canadians’ knowledge of cancer risk factors and belief in cancer myths

**DOI:** 10.1186/s12889-025-22185-6

**Published:** 2025-03-10

**Authors:** E Rydz, J Telfer, EK Quinn, SS Fazel, E Holmes, G Pennycook, CE Peters

**Affiliations:** 1 School of Population and Public Health, CAREX Canada, University of British Columbia, Vancouver, EK Canada; 2 Department of Oncology, Cumming School of Medicine, University of Calgary, Calgary, Canada; 3 Canadian Cancer Society, Toronto, Canada; 4 Department of Psychology, Cornell University, New York, CE USA; 5 BC Centre for Disease Control, Vancouver, BC Canada; 6 BC Cancer, Vancouver, BC Canada

**BMC Public Health (2024) 24:329.**

10.1186/s12889-024-17832-3.

In the original publication of this article the captions of Figs. 2 and 3 were swapped. The incorrect and correct information is listed in this correction article. The original article is updated.

Incorrect.

Figure 2 Percent breakdown of responses to “to what extent do you agree that the following can reduce cancer risk”? (*n* ~ 400 per factor) *Known protective factors.

Figure 3 Percent breakdown of responses to “to what extent do you agree that the following can increase cancer risk”? (*n* ~ 400 per factor) *Known risk factors.

Correct.

Figure 2 Percent breakdown of responses to “to what extent do you agree that the following can increase cancer risk”? (*n* ~ 400 per factor) *Known risk factors.

Figure 3 Percent breakdown of responses to “to what extent do you agree that the following can reduce cancer risk”? (*n* ~ 400 per factor) *Known protective factors.

