# Supplementary material for: Canadians’ knowledge of cancer risk factors and belief in cancer myths
Source: BMC Public Health. 2024 Jan 30;24:329. doi: 10.1186/s12889-024-17832-3 (PMC10829248; doi:10.1186/s12889-024-17832-3)
Supplement: Supplementary file 1 — Supplementary Material 1: Additional file 1 [file 12889_2024_17832_MOESM1_ESM.docx]

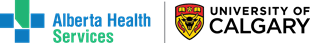


**Assessing Canadians' awareness of risk factors and belief in myths about what causes cancer.**

## Project background and consent

*[This section* ***will be the first page of the online survey. Only after participants click, ‘proceed to questionnaire’ will they be given access to the survey questions.]***

**Researchers:**

Dr. Cheryl Peters ([cheryl.peters@ucalgary.ca](mailto:cheryl.peters@ucalgary.ca))

**Background & Purpose:**The aim of this study is to help researchers understand what cancer risk factors Canadians are aware of, what myths about the causes of cancer are most commonly believed, and how different thinking styles may impact beliefs.

**Why are we doing this research?**

This is the first study conducted in Canada which helps to understand Canadians’ awareness about the causes of cancer and myths related to the causes of cancer. The results of this study will be very important in helping to create information materials about the risks of cancer and debunking cancer myths.

**Who is eligible to take part?**

Adults aged 18+ and residents of Canada are invited to participate in this study.

**What happens if I decide to take part in this study?**

If you agree to take part, you will be directed to an online survey. The survey asks questions about your knowledge and beliefs regarding the causes of cancer. The survey also includes some general questions about you (your age group, education, gender, etc), and about your thinking style. We estimate that it will take 15 minutes to complete the survey.

**How will my personal information be kept private?**

If you decide to participate, the researcher and study team will only collect information they need for this study. They will do everything that they can to make sure that this data is kept private/confidential. All information collected for data analysis and otherwise will be accessed on a password protected computer, and stored on a secure hard drive. Survey data will also be stored on Qualtrics servers located in Canada and your confidentiality will be maintained.

**Are there any benefits to participation?**

Participation in this study is voluntary and by participating in this study, you are helping us understand the current knowledge and beliefs among Canadians about the causes of cancer.

**Are there any risks to participation?**

There are no foreseeable risks associated with your participation in this research.

**Can I withdraw from this study at any stage?**

You can choose to end your participation in this research study at any stage by exiting from the survey. Information that was recorded before you pressed ‘submit’ will be used by the researcher for this study, but no additional information will be collected.

**Who do I contact for questions related to this study?**

For more information you can contact the study coordinator at [erydz@carexcanada.ca](mailto:erydz@carexcanada.ca) or the lead researcher at [cheryl.peters@ucalgary.ca](mailto:cheryl.peters@ucalgary.ca)

**Consent**

By clicking ‘Proceed to questionnaire’ below you will be agreeing to the following:

- I confirm that I have read and understood the information presented about the research and that I am aware of the nature, purpose, and duration of the study.
- I have had time to consider whether I want to take part in this study.
- I understand that I am free to withdraw at any time while I am completing the survey.
- I understand that the data will be presented and/or published in academic journals and at conferences, but that no participant will be identifiable from the information.

[Proceed to questionnaire button]

[Do not proceed button]

**Questionnaire**

## Causes of Cancer

1. For the following statements, how much do you agree that each of these diet-related factors can **increase** a person’s chance of developing cancer? (Options: Strongly disagree, Disagree, Neither agree nor disagree, Agree, Strongly agree)
   1. Eating food containing additives
   2. Drinking coffee once a day or more
   3. Eating less than 5 portions of vegetables and fruit a day
   4. Eating food containing artificial sweeteners
   5. Consuming more than 1 alcoholic drink a day
   6. Eating genetically modified food (‘GMOs’)
   7. Eating red or processed meat once a day or more
   8. Drinking from plastic bottles
   9. Eating foods containing any type of sugar
   10. Drinking fluoridated water once a day or more
2. For the following statements, how much do you agree that each of these can **increase** a person’s chance of developing cancer? (Options: Strongly disagree, Disagree, Neither agree nor disagree, Agree, Strongly agree)
   1. Smoking cigarettes
   2. Sustaining physical injury or trauma
   3. Using cell phones
   4. Feeling stressed
   5. Being exposed to another person’s cigarette smoke
   6. Being exposed to 5G and Wi-Fi signals
   7. Being over 70 years old
   8. Having negative thoughts
   9. Using microwave ovens once a day or more
   10. Having a close relative with cancer
   11. Wearing a bra
   12. Doing less than 30 mins of moderate physical activity 5 times a week
   13. Having an HPV (human papillomavirus) infection
   14. Using antiperspirants or deodorants once a day or more
   15. Getting vaccinated
   16. Getting sunburnt more than once
   17. Using sunscreen
   18. Living near power lines
   19. Using hair dyes
   20. Interacting with someone who has cancer
   21. Being exposed to electromagnetic frequencies
   22. Being exposed to glyphosate
   23. Using toiletries or cosmetics
   24. Having bad luck
3. For the following statements, how much do you agree that each of these can **reduce** a person’s chance of developing cancer? (Options: Strongly disagree, Disagree, Neither agree nor disagree, Agree, Strongly agree)
   1. Eating organic foods
   2. Exercising for at least 30 mins for 5 times a week
   3. Eating 5 portions or more of vegetables and fruit a day
   4. Reducing exposure to radon at home
   5. Having a base tan
   6. Reducing consumption of red or processed meat
   7. Taking vitamins, supplements or herbal products once a day
   8. Eating superfoods rich in antioxidants
   9. Drinking red wine
   10. Living smoke-free
   11. Eating an alkaline diet
   12. Practicing sun safety (e.g. covering up, or wearing a hat, sunglasses, or sunscreen)
   13. Getting the HPV vaccine
   14. Consuming cannabis
   15. Maintaining a healthy body weight
   16. Protecting yourself from hazardous substances at work

## Thinking styles

Please indicate the extent to which you agree or disagree with the following statements. (Options: Strongly disagree, Disagree, Neither agree nor disagree, Agree, Strongly agree)

1. It is important to be loyal to your beliefs even when evidence is brought to bear against them.
2. Whether something feels true is more important than evidence.
3. Just because evidence conflicts with my current beliefs does not mean my beliefs are wrong.
4. There may be evidence that goes against what you believe but that does not mean you have to change your beliefs.
5. Even if there is concrete evidence against what you believe to be true, it is OK to maintain cherished beliefs.
6. Regardless of the topic, what you believe to be true is more important than evidence against your beliefs.
7. I think there are many wrong ways, but only one right way, to almost anything.
8. In my experience, the truth is often black and white.
9. Truth is never relative.
10. The truth does not change.
11. Either something is true or it is false; there is nothing in-between.
12. There is no middle ground between what is true and what is false.

**Thinking styles continued…**

Please indicate the extent to which you agree or disagree with the following statements. (Options: Strongly disagree, Disagree, Neither agree nor disagree, Agree, Strongly agree)

1. I like to rely on my intuitive impressions.
2. I believe in trusting my hunches.
3. When I make decisions, I tend to rely on my intuition.
4. Using my "gut-feelings" usually works well for me in figuring out problems in my life.
5. Intuition is the best guide in making decisions.
6. I often go by my instincts when deciding on a course of action.
7. I’m not that good at figuring out complicated problems.
8. Thinking is not my idea of an enjoyable activity.
9. I try to avoid situations that require thinking in depth about something.
10. I am not a very analytical thinker.
11. Reasoning things out carefully is not one of my strong points.
12. Thinking hard and for a long time about something gives me little satisfaction.
13. I am very busy these days and do not have time to follow the latest research. To show that you’re still paying attention, answer both “strongly disagree” and “disagree”. [Allow user to select two responses for this question]

## Background information

1. What province or territory do you live in? (Dropdown menu)
   1. Alberta
   2. British Columbia
   3. Manitoba
   4. New Brunswick
   5. Newfoundland and Labrador
   6. Northwest Territories
   7. Nova Scotia
   8. Nunavut
   9. Ontario
   10. Prince Edward Island
   11. Quebec
   12. Saskatchewan
   13. Yukon
   14. Prefer not to say
2. How old are you? (Free text) ________
3. What is your gender? (Select from list)
   1. Man
   2. Woman
   3. Non-binary or third gender
   4. Two-spirit
   5. Prefer not to say
   6. Prefer to self-describe:
4. Which one of the following describes where you live? (Select from list)
   1. Rural (population of less than 50,000)
   2. Small town (population from 50,000 to <250,000)
   3. Large city (population from 250,000 to <1 million)
   4. Metropolitan center (population of 1 million or more)
   5. Prefer not to say
5. What is your racial or ethnic background (Select all that apply)?
   1. Arab
   2. Black
   3. Chinese
   4. Filipino
   5. First Nations
   6. Inuit
   7. Japanese
   8. Korean
   9. Latin American
   10. Métis
   11. South Asian
   12. South East Asian
   13. White
   14. West Asian
   15. Prefer not to say
   16. Other, please specify:
6. What is your total annual household income? (Dropdown menu)
   1. Less than $20,000
   2. $20,000 to $39,999
   3. $40,000 to $59,999
   4. $60,000 to $79,999
   5. $80,000 to $99,999
   6. $100,000 to $119,999
   7. $120,000 or more
   8. Prefer not to say
7. Which one of the following statements best describes you? (Select from list)
8. I am not a person living with cancer or a caregiver for someone who has been diagnosed with cancer
9. I am caring for someone who has been diagnosed with cancer
10. I was recently diagnosed with cancer and I am waiting to start my treatment
11. I am currently undergoing treatment for my cancer (this is any treatment including chemotherapy, radiation and surgery)
12. My cancer has been treated and I continue to have follow-up appointments with my doctor
13. My cancer has been treated and I no longer have appointments with my doctor
14. I have been diagnosed with advanced cancer
15. I am currently receiving end-of-life care

Thank you for completing this survey. Your feedback is very valuable. To learn more about what you can do to reduce your cancer risk, visit the Canadian Cancer Society at [www.cancer.ca/prevention](http://www.cancer.ca/prevention).
