# Supplementary material for: Canadians’ knowledge of cancer risk factors and belief in cancer myths
Source: BMC Public Health. 2024 Jan 30;24:329. doi: 10.1186/s12889-024-17832-3 (PMC10829248; doi:10.1186/s12889-024-17832-3)
Supplement: Supplementary file 2 — Supplementary Material 2: Additional file 2 [file 12889_2024_17832_MOESM2_ESM.docx]

## Additional file 3. Spearman correlations between thinking disposition scores and agreement that select factors increase cancer risk

|  | AOT score | PET score | PIT score | CMT score | Overall % correctly identified |
| --- | --- | --- | --- | --- | --- |
| Eat less than five portions of vegetables or fruit per day | 0.042 | -0.042 | -0.078 | 0.024 | 68.6 |
| Drink more than one alcoholic drink per day | 0.065 | 0.018 | -0.066 | 0.040 | 54.5 |
| Eat red or processed meat once a day or more | 0.162* | 0.074 | -0.145* | -0.022 | 53.0 |
| Smoke cigarettes | 0.153* | 0.161* | -0.064 | -0.190** | 97.8 |
| Exposed to SHS | 0.188** | 0.169* | -0.094 | -0.157* | 92.6 |
| Be 70 Years or older | 0.173** | 0.206** | -0.152* | -0.040 | 60.5 |
| Have a close relative with cancer | 0.137* | 0.125* | -0.105* | -0.094 |  |
| Do less than 30 minutes of physical activity 5 times a week | 0.094 | -0.002 | -0.059 | -0.045 | 74.6 |
| Have HPV infection | 0.166* | 0.156* | -0.048 | -0.133* | 59.7 |
| Get sunburnt | 0.122* | 0.188** | -0.030 | -0.210** | 82.8 |
| Use sunscreen | -0.170* | -0.061 | 0.136* | 0.145* | 82.5 |
| Consume additives | -0.123* | 0.056 | 0.212** | 0.093 | 15.9 |
| Drink coffee | -0.112* | -0.152* | 0.168* | 0.113* | 67.5 |
| Have bad luck | -0.114* | -0.143* | 0.018 | 0.055 | 65.1 |
| Consume artificial sweeteners | -0.017 | 0.036 | 0.123* | 0.070 | 23.3 |
| Eat GMO foods | -0.362** | -0.155* | 0.284** | 0.166* | 44.0 |
| Drink from plastic bottles | 0.008 | 0.168* | 0.061 | -0.062 | 34.1 |
| Eat foods with sugar | -0.071 | 0.047 | 0.077 | 0.140* | 44.6 |
| Drink fluoridated water once a day or more | -0.264** | -0.226** | 0.227** | 0.117* | 57.0 |
| Sustain physical injuries and trauma | -0.067 | -0.108* | -0.030 | 0.036 | 50.8 |
| Use cellphones | -0.292** | 0.011 | 0.256** | 0.195** | 49.3 |
| Feel stressed | -0.024 | 0.046 | 0.011 | -0.126* | 25.6 |
| Exposed to 5G and WiFi | -0.270** | -0.168* | 0.245** | 0.178** | 70.4 |
| Have negative thoughts | -0.145* | -0.127* | 0.063 | 0.058 | 66.3 |
| Use microwaves once a day or more | -0.232** | -0.100 | 0.226** | 0.189** | 63.8 |
| Wear a bra | -0.114* | -0.163* | 0.099 | 0.044 | 78.0 |
| Use deodorants or antiperspirants once a day or more | -0.102* | -0.001 | 0.100 | 0.023 | 51.2 |
| Get vaccinated | -0.248** | -0.162* | 0.197** | 0.198** | 87.5 |
| Live near power lines | -0.273** | -0.114* | 0.249** | 0.175** | 52.5 |
| Use hair dyes | -0.145* | -0.109* | 0.066 | 0.106* | 47.1 |
| Interact with someone with cancer | -0.231** | -0.184** | 0.055 | 0.145* | 98.1 |
| Be exposed to electromagnetic frequencies | -0.283** | -0.056 | 0.190** | 0.137* | 31.0 |
| Be exposed to glyphosate | -0.095 | -0.022 | 0.035 | 0.062 | 8.5 |
| Use toiletries or cosmetics | -0.169* | -0.064 | 0.109* | 0.077 | 51.8 |

**Known risk factors are bolded**

***p-value<0.05; **p-value<0.001**
