# Supplementary material for: Canadians’ knowledge of cancer risk factors and belief in cancer myths
Source: BMC Public Health. 2024 Jan 30;24:329. doi: 10.1186/s12889-024-17832-3 (PMC10829248; doi:10.1186/s12889-024-17832-3)
Supplement: Supplementary file 3 — Supplementary Material 3: Additional file 3 [file 12889_2024_17832_MOESM3_ESM.docx]

## Additional file 2. Pearson correlations between thinking styles.

|  | AOT score | PET score | PIT score | CMT score |
| --- | --- | --- | --- | --- |
| AOT score | - |  |  |  |
| PET score | 0.37995** | - |  |  |
| PIT score | -0.49584** | -0.29166** | - |  |
| CMT_score | -0.30976** | -0.23939** | 0.22288** | - |

**p<0.001
