# Supplementary material for: Canadians’ knowledge of cancer risk factors and belief in cancer myths
Source: BMC Public Health. 2024 Jan 30;24:329. doi: 10.1186/s12889-024-17832-3 (PMC10829248; doi:10.1186/s12889-024-17832-3)
Supplement: Supplementary file 4 — Supplementary Material 4: Additional file 4 [file 12889_2024_17832_MOESM4_ESM.docx]

## Additional file 4. Spearman correlations between thinking disposition scores and agreement that select factors reduce cancer risk

|  | AOT score | PET score | PIT score | CMT score | Overall % correctly identified |
| --- | --- | --- | --- | --- | --- |
| Exercise at least 30 minutes, 5 times a week | -0.002 | 0.083 | -0.081 | -0.070 | 76.4 |
| Maintain a health body weight | -0.048 | 0.093 | -0.023 | 0.047 | 80.7 |
| Get the HPV vaccine | 0.254** | 0.187** | -0.145* | -0.213** | 59.7 |
| Reduce exposure to radon at home | 0.030 | 0.157* | -0.008 | 0.015 | 69.3 |
| Reduce red or processed meat consumption | 0.130* | 0.045 | -0.109* | -0.107* | 64.5 |
| Live smoke-free | 0.218** | 0.227** | -0.142* | -0.171* | 96.7 |
| Practise sun safety | 0.230** | 0.167* | -0.073 | -0.200** | .96.5 |
| Eat 5 portions or more of vegetables and fruits a day | 0.048 | 0.029 | -0.073 | -0.049 | 23.4 |
| Protect yourself from hazardous substances at work | 0.200** | 0.190** | -0.101 | -0.039 | 94.9 |
| Drink red wine | -0.000 | -0.064 | 0.005 | 0.055 | 43.7 |
| Eat an alkaline diet | -0.170* | -0.189** | 0.256** | 0.106* | 31.5 |
| Have a base tan | -0.252** | -0.237** | 0.150* | 0.223** | 66.6 |
| Consume cannabis | -0.113* | -0.081 | 0.189** | 0.073 | 61.3 |
| Eat organic foods | -0.240** | -0.124* | 0.211** | 0.146* | 27.8 |
| Eat superfoods rich in antioxidants | -0.108* | -0.047 | 0.185** | 0.099 | 11.4 |
| Take vitamins, supplements, or herbal products once a day | -0.255** | -0.147* | 0.213** | 0.160* | 24.5 |

**Known risk factors are bolded**

***p-value<0.05; **p-value<0.001**
